# Hospital-service use in the last year of life by patients aged ⩾60 years who died of heart failure or cardiomyopathy: A retrospective linked data study

**DOI:** 10.1177/02692163231180912

**Published:** 2023-06-12

**Authors:** Gursharan K Singh, Alison P Bowers, Caleb Ferguson, Serra E Ivynian, Shirley Chambers, Patricia M Davidson, Louise D Hickman

**Affiliations:** 1 Centre for Healthcare Transformation, Faculty of Health, Queensland University of Technology (QUT), Brisbane, QLD, Australia; 2 Cancer and Palliative Care Outcomes Centre, School of Nursing, Queensland University of Technology (QUT), Brisbane, QLD, Australia; 3 University of Wollongong, Wollongong, NSW, Australia; 4 IMPACCT, Faculty of Health, University of Technology Sydney, Ultimo, Sydney, NSW, Australia

**Keywords:** Heart failure, hospitalisation, end-of-life care, palliative care, hospitals

## Abstract

**Background:**

Understanding patterns of health care use in the last year of life is critical in health services planning.

**Aim:**

To describe hospital-based service and palliative care use in hospital in the year preceding death for patients who died of heart failure or cardiomyopathy in Queensland from 2008 to 2018 and had at least one hospitalisation in the year preceding death.

**Design:**

A retrospective data linkage study was conducted using administrative health data relating to hospitalisations, emergency department visits and deaths.

**Participants and setting:**

Participants included were those aged ⩾60 years, had a hospitalisation in their last year of life and died of heart failure or cardiomyopathy in Queensland, Australia.

**Results:**

Of the 4697 participants, there were 25,583 hospital admissions. Three quarters (*n* = 3420, 73%) of participants were aged ⩾80 years and over half died in hospital (*n* = 2886, 61%). The median number of hospital admissions in the last year of life was 3 (interquartile range [IQR] 2–5). The care type was recorded as ‘acute’ for 89% (*n* = 22,729) of hospital admissions, and few (*n* = 853, 3%) hospital admissions had a care type recorded as ‘palliative.’ Of the 4697 participants, 3458 had emergency department visit(s), presenting 10,330 times collectively.

**Conclusion:**

In this study, patients who died of heart failure or cardiomyopathy were predominantly aged ⩾80 years and over half died in hospital. These patients experienced repeat acute hospitalisations in the year preceding death. Improving timely access to palliative care services in the outpatient or community setting is needed for patients with heart failure.

**What is already known about the topic?**

- Evidence based guidelines recommend palliative care for individuals with advanced heart failure, yet there is limited data on acute hospital and palliative care service use prior to death, to inform care planning and end-of-life services.

**What this article adds?**

- Patients were generally aged ⩾80 years old and experienced repeat acute hospitalisations in the year preceding death.
- Over half died in hospital, without a hospitalisation for palliative care recorded.
- For those who had a hospitalisation for palliative care recorded, this mostly occurred in the last 7 days of life.

**Implications for practice, theory of policy**

- Improving access to palliative care services in the outpatient or community setting is needed to reduce avoidable hospitalisations.
- Improving access to timely palliative care, including end-of-life care, for patients with heart failure is needed.

## Introduction

The global prevalence of heart failure is increasing with the rising ageing population.^1^ Heart failure is characterised by severe symptom burden, poor quality of life and frequent hospitalisation.^2^ Heart failure is the most common cardiovascular reason for hospitalisation among patients aged ⩾60 years.^2^

The illness trajectory of heart failure is commonly characterised as a dynamic and cyclical process of acute decompensation, deterioration and recovery and is associated with increased acute health service use and socio-economic burden.^3^ Individuals living with serious illnesses including heart failure, are more likely to use hospital-based services during the last year of life and as death approaches.^4,5^ This is a time where needs assessments and palliative care provision is essential^6^ and evidence-based guidelines recommend palliative care for individuals with advanced heart failure.^7^ Specialist palliative care can improve symptom management and reduce acute health service use.^8^ However, the illness trajectory of heart failure which is longer and more unpredictable than cancer complicates the provision of specialist palliative care.^9^

Population-level data describing hospital-based service and palliative care use in the last year of life by people who died of heart failure or cardiomyopathy is critical to plan health services, workforce and care provision which enables improved access to palliative care.^9^ There is currently limited information relating to palliative care use in the last year of life for individuals who died of heart failure or cardiomyopathy and referral and uptake is highly variable. Furthermore, it is essential to take into account the healthcare use patterns within particular healthcare jurisdictions while designing and assessing models of care for distinct clinical diagnoses.

## Aim

The aim of this study was to describe the use of acute hospital-based health services by patients aged ⩾60 years who died of heart failure or cardiomyopathy and had a hospital episode in their last year of life. Specifically, we sought to examine patterns of hospital admissions, including admissions to an intensive care unit (ICU), comorbidities and palliative care hospital admissions as well as emergency department presentations.

## Methods

### Data sources and case record linkage

This study was a retrospective, population-based linkage study utilising the Queensland Hospital Admitted Patient Data Collection, Queensland Emergency Department Data Collection and the Queensland Death Registration Data. The datasets were linked by a unique patient identification number and included data from 1st July 2008 to 31st December 2018 (the most recently available data for which death information was available), to capture as many individuals as possible. Data for this study were extracted by the Queensland Health Statistical Services Branch in February 2021 and was provided as four separate data sets.

### Setting

Queensland Health in Australia plays a key role in managing the State’s public hospitals and health services and comprises of the Department of Health and 16 separate Hospital and Health Services (HHSs).^10^ The Department of Health is responsible for managing the public health system in Queensland, while the HHSs manage community health services within their district.^10^

### Sample

All patients aged ⩾60 years, who died in Queensland of heart failure or cardiomyopathy and had a hospital episode in the last year of life were included. Patients aged ⩾60 years were selected due to this being the age at which prevalence of heart failure rises and hospitalisations sharply increase.^2^ Patients who died of cardiomyopathy were included based on the recommendation that studies utilising administrative data for heart failure should also consider codes for cardiomyopathies.^11^ The underlying cause of death as identified by Cause of Death Unit Record File data was recorded using the International Statistical Classification of Diseases and Related Health Problems 10th revision (ICD-10) diagnoses: I50 and I42, including all subcodes.

### Data collection

The variables of interest included the number and length of stay of hospital admissions and emergency department visits, time spent in an ICU in proximity to death and ‘died in hospital’ as the mode of separation. Comorbidities, as listed in the Queensland Hospital Admitted Patient Data Collection are also examined and includes any additional disease or injury occurring alongside other disease and injury. A measure of interest, in proximity to death, was the proportion of hospital admission episodes recorded in the Queensland Hospital Admitted Patient Data Collection with a ‘palliative’ care type in the last 12 months of life. Care type is the nature of care or treatment provided during hospitalisation. ‘Palliative’ care type is when the nature of care or treatment is focussed on optimising quality of life, is delivered by health professionals with specialist palliative care training, that can be, but does not necessarily need to be, delivered in a designated palliative care unit or when the management plan and clinical intent are informed in consultation with a specialist palliative care clinician and delivered by another medical team. Previous specialist non-admitted service contact for palliative care is also collected from Queensland Hospital Admitted Patient Data Collection and includes patients seen at any time in the past for non-admitted specialist palliative care treatment in an outpatient service or a community health service.

### Data analysis

All data were analysed using SPSS Version 26 (IBM Corp., Armonk, NY, USA). To determine the median number of admissions and median length of stay, hospital episodes with overlapping dates that arose from changes in care type, were aggregated to represent one hospital admission. As the proportion of hospital admission episodes recorded in the Queensland Hospital Admitted Patient Data Collection with a ‘palliative’ care type were a measure of interest, the care type was not investigated at an aggregated level. Characteristics were analysed using descriptive statistics. To determine potential differences in demographic characteristics between patients who had a record of a palliative care episode, chi-squared test was used for categorical data.

### Ethics

This study was approved by Queensland University of Technology Human Research Ethics Committee (Ref 2000000636) in September 2020, prior to the commencement of the study. All data was kept confidential and stored securely and small numbers were aggregated to ensure anonymity.

## Results

### Patient characteristics

There was a total of 4697 patients aged ⩾60 years who died of heart failure or cardiomyopathy and were hospitalised in their last year of life. There were approximately equal numbers of males (*n* = 2266, 48%) and females (*n* = 2431, 52%) and most (*n* = 3420, 73%) were aged ⩾80 years (Table 1).

**Table 1.** Characteristics of patients aged ⩾60 years who died of heart failure or cardiomyopathy and had a hospital episode in the final year of life.

Characteristics	*n* (%)
Sex^a^	
Female	2431 (52)
Male	2266 (48)
Cause of death	
ICD-10 diagnosis: I42 cardiomyopathy	1422 (30)
ICD-10 diagnosis: I50 heart failure	3275 (70)
Age (years)^a^	
60–64	200 (4)
65–69	231 (5)
70–74	345 (7)
75–79	501 (11)
80–84	873 (19)
85+	2547 (54)
Marital status^a,b^	
Not partnered	2561 (56)
Partnered	1981 (44)
Country of birth^a,c^	
Australia	3559 (76)
Europe	788 (17)
New Zealand	117 (2)
Asia	74 (2)
Africa and Middle East	41 (1)
Oceania and Antarctica	33 (1)
America	29 (1)
ARIA + status (remoteness of home location)^a^	
Major city	2658 (57)
Outer regional	615 (13)
Inner regional	1242 (26)
Very remote	42 (1)
Remote	59 (1)
Other/outside of Queensland	81 (2)
Socioeconomic status^a,d^	
Most disadvantaged quintile	1207 (26)
Quintile 2	1055 (23)
Quintile 3	956 (21)
Quintile 4	728 (16)
Least disadvantaged quintile	669 (14)

ICD-10: International Statistical Classification of Diseases and Related Health Problems 10th revision.

a At last episode recorded in the last year of life.

b *n* = 4542.

c *n* = 4641.^d^*n* = 4615, using population-based quintiles of the Australian Bureau of Statistics’ index of relative disadvantage.

### Hospital admissions

**Figure 1.**
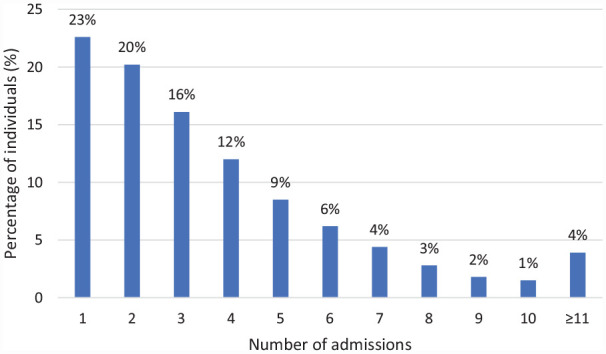
Admissions in the last year of life (*n* = 4697).

Of the 4697 patients, 2886 (61%) died in hospital and the median length of stay of the hospital admission resulting in death was 6 days (interquartile range [IQR] 2–15), compared to a median length of 2 days (IQR 1–8) for all hospital admissions. The median number of hospital admissions in the last year of life was 3 (IQR 2–5) excluding same day admissions recorded as haemodialysis (Figure 1). There were 1062 (23%) patients who did not have repeat admissions. In terms of proximity to death, 2058 (44%) patients had a hospital admission in the last 7 days of life, 3409 (73%) in the last 30 days of life and 4062 (86%) in the last 90 days of life.

Table 2 provides information on the total 25,583 hospital admissions. The majority (*n* = 22,729, 89%) of hospital admissions had a care type recorded as ‘acute,’ while very few (*n* = 853, 3%) hospital admissions had a care type recorded as ‘palliative.’ Almost half (*n* = 10,679, 42%) of hospital admissions were from the emergency department and more than half of the hospital admissions (*n* = 17,455, 68%) ended in the individual being discharged to their home or usual residence.

**Table 2.** Hospital admissions in the final year of life for patients aged ⩾60 years who died of heart failure or cardiomyopathy (*N* = 25,583).

Admission information	*n* (%)
Care type	
Acute	22,729 (89)
Rehabilitation	990 (4)
Maintenance	649 (3)
Palliative care	853 (3)
Geriatric evaluation and management	338 (1)
Mental health and other care	24 (0.1)
Elective status	
Emergency admission	12,527 (49)
Not assigned	9174 (36)
Elective admission	3882 (15)
Source of referral	
Emergency department – this hospital	10,679 (42)
Routine readmission – not requiring referral	5291 (21)
Private medical practitioner	3741 (14)
Episode Change	2289 (9)
Patient transferred from another hospital	1985 (8)
Outpatient department – this hospital	987 (4)
Residential aged care service	367 (1)
Other healthcare establishment	216 (1)
Community service	28 (0.1)
Mode of separation	
Home/usual residence	17,455 (68)
Died in hospital	2886 (11)
Care type change	2249 (9)
Transferred to another hospital	2020 (8)
Residential aged care service	662 (3)
Other healthcare establishment	311 (1)

### Admissions to ICU

There were 167 (4%) patients who had an ICU admission associated with hospitalisation. The 167 patients collectively had a total of 190 hospital admissions with an admission to the ICU and 147 (88%) patients had only one ICU admission. Of the 167 patients, 36 (22%) had an ICU admission in the last 7 days of life, 86 (51%) in the last 30 days and 116 (67%) in the last 90 days of life. There were 72 patients who died in hospital during an admission with admission to ICU, although the data did not indicate if the death occurred while in the ICU.

### Comorbidities

Of the 4697 patients, 4694 (99%) reported comorbidity data. The 4694 patients had a median of 4 comorbidities (IQR 2–6) recorded at their first admission in their last year of life. Heart failure or cardiomyopathy were recorded for 1938 patients (41%), followed by symptoms, signs and abnormal clinical findings (*n* = 1822, 39%), diseases of the respiratory system (*n* = 1437, 31%), hypertension (*n* = 1386, 30%) and endocrine, nutritional and metabolic diseases (*n* = 1338, 29%).

### Hospital admissions for palliative care

There were 768 (16%) patients who had a hospital admission care type recorded as ‘palliative’, and 70% (*n* = 537) were aged ⩾80 years, with equal proportion of males (*n* = 385, 50%) and females (*n* = 383, 50%). These 768 patients had a median of 5 hospital admissions (IQR 3–7) in the last year of life when a palliative care type admission was documented, with the median length of stay for palliative care admission being 3 days (IQR 1–6). There were 44 (6%) patients who had a record for a previous admission for palliative care treatment and 78 (10%) patients who had a record for previous non-admitted contact for palliative care treatment. A total of 676 (88%) patients had their separation documented as ‘died in hospital’ during a palliative care type admission. In terms of proximity to death, 550 (72%) patients had a palliative care type admission recorded in the last 7 days of life, 690 (89%) in the last 30 days and 738 (96%) patients in the last 90 days of life. There were 30 (4%) patients who had a palliative care admission recorded >90 days before death.

The group of patients with a record of a palliative care hospitalisation had a higher proportion of individuals aged 70–74 (11% versus 7%, *p* = 0.002) and a higher proportion of partnered individuals (46% versus 41%, *p* = 0.025). There was a higher proportion of patients who were not born in Australia, compared to those who were (29% versus 22% respectively, *p* < 0.001) and a lower proportion of patients who resided in very remote, remote and areas outside Queensland (2% versus 4%, *p* = 0.014) in the group with a record of a palliative care hospitalisation (Table 3).

**Table 3.** Characteristics of patients aged ⩾60 years who died of heart failure or cardiomyopathy with a record of hospital-based palliative care admissions in the last year of life.

	Did not have a record of hospital-based palliative care	Did have a record of hospital-based palliative care	*p* value
	*n* (%)	n (%)	
Age (years)^a^			0.002
60–64	169 (4)	31 (4)	
65–69	200 (5)	31 (4)	
70–74	263 (7)	82 (11)	
75–79	414 (11)	87 (11)	
80–84	723 (18)	150 (20)	
85+	2160 (55)	387 (50)	
Total	3929 (100)	768 (100)	
Sex^a^			0.253
Female	2048 (52)	383 (50)	
Male	1881 (48)	385 (50)	
Total	3929 (100)	768 (100)	
Marital status^a^			0.025
Not partnered	2300 (59)	416 (54)	
Partnered	1629 (41)	352 (46)	
Total	3929 (100)	768 (100)	
Country of birth^a,b^			<0.001
Australia	3024 (78)	535 (71)	
Not Australia	859 (22)	223 (29)	
Total	3883 (100)	758 (100)	
ARIA+ Status (remoteness of home location)^a^			0.014
Inner regional	1053 (27)	189 (25)	
Major city	2198 (56)	460 (60)	
Outer regional	512 (13)	103 (13)	
Very remote, remote and outside QLD	166 (4)	16 (2)	
Total	3929 (100)	768 (100)	
Socioeconomic status^a,c^			0.733
Most disadvantaged quintile	1004 (26)	203 (27)	
Quintile 2	889 (23)	166 (22)	
Quintile 3	803 (21)	153 (20)	
Quintile 4	597 (16)	131 (17)	
Least disadvantaged quintile	562 (15)	107 (14)	
*Total*	3855 (100)	760 (100)	

a At last episode recorded in the last year of life.

b *n* = 4641.

c *n* = 4615 using population-based quintiles of the Australian Bureau of Statistics’ index of relative disadvantage.

### Emergency presentations

Of the 4697 patients who had a hospital episode, 3458 (74%) had an emergency department presentation, totalling 10,330 emergency department presentations. The median number of emergency presentations in the last year of life was 2 (IQR 1–4). There were 1179 (34%) patients who presented to the emergency department in the last 7 days, 2253 (65%) who presented in the last 30 days of life and 2878 (83%) who presented in the last 90 days of life. Table 4 presents information relating to the emergency department presentations and shows over half (*n* = 6819, 66%) of emergency department presentations were by patients aged ⩾80 years and the majority (*n* = 8025, 79%) arrived by ambulance.

**Table 4.** Emergency presentations in the last year of life by patients aged ⩾60 years who died of heart failure or cardiomyopathy (*N* = 10,330).

Emergency presentation information	*n* (%)
Sex	
Female	4915 (48)
Male	5415 (52)
Age (years)	
55–59	21 (0.2)
60–64	517 (5)
65–69	644 (6)
70–74	932 (9)
75–79	1397 (14)
80–84	2023 (20)
85+	4796 (46)
Mode of transport by which patient arrives at the ED^a^	
Ambulance	8025 (79)
Self-presented	2246 (21)
Top 5 principal diagnoses^b^	
Heart failure	1919 (19)
Diseases of the respiratory system	1395 (14)
Injury, poisoning and other consequences of external causes	1345 (13)
Symptoms, signs and abnormal clinical laboratory findings	1275 (12)
Diseases of the circulatory system	1169 (11)
Reason for patient presenting to the ED	
Emergency presentation	10032 (97)
Planned return visit	143 (1)
Transfer-in	55 (0.5)
Inter-hospital transfer	43 (0.4)
Unplanned return for the current condition	35 (0.3)
Pre-arranged admission	22 (0.2)
Status of the patient at end of ED service	
Admitted	7560 (73)
ED service event completed – discharged	1954 (19)
Transferred to another hospital	601 (6)
Died in ED or on arrival	135 (1)
Did not wait	80 (1)
Triage code	
1 = Immediately life threatening	395 (4)
2 = Imminently life threatening	2398 (23)
3 = Potentially life threatening	5415 (52)
4 = Potentially serious	1780 (17)
5 = Non-urgent	342 (3)

ED: emergency department.

a *n* = 10,271.

b *n* = 7103.

## Discussion

In this study, participants were generally aged ⩾80 years old, experienced repeat acute hospitalisations in the year preceding death and dying in hospital was common. Despite this, there was limited recording of access to palliative care. This is reflective of findings of a similar study describing health care use in the last year of life.^5^

Compared to younger patients, older patients with heart failure (aged ⩾80 years) have a different clinical profile, often with complex comorbidities, leading to rehospitalisation and progressive decline.^12^ The participants in this study had a median of four comorbidities. Patients with heart failure who are older and have comorbidities may experience adverse events which lead to preventable hospitalisations.^13^ This may be due to an underuse of effective heart failure therapies in the presence of other comorbidities due to safety concerns and difficulty recalling complex medication regimens,^13^ as older patients with heart failure and their carers may experience challenges related to frailty, functional decline and extensive self-care requirements of heart failure management.^14^ The provision and availability of multidisciplinary, specialised heart failure services that provide follow-up, evidence-based care and education of patients to enhance self-care is essential to address the issue of comorbidities and complex medication regimens.^15^ Timely access to evidence-based, multidisciplinary heart failure services is cost-neutral or cost-saving and is associated with reduced preventable hospital admissions, improved adherence to evidence-based treatment and enhanced quality of life.^15^ However, greater resources and funding must be put in place to enable timely access to heart failure care.^16^ Integration of community and secondary care services, and multidisciplinary collaboration with primary care and nurse-led heart failure care is needed.^16^

In this study, more than half of the patients died in hospital. While studies have demonstrated that over 60% of patients prefer to die at home,^17,18^ other studies have found patients with poor self-rated health and/or more chronic diseases were likely to prefer to die in a healthcare facility as opposed to home, arguably because they understand the realities of home care better than patients with good self-rated health.^19^ Identifying and achieving preferred place of death is important and can be achieved through high-quality advance care planning.^20^ Advance care planning includes preparing patients and their surrogate decision maker(s) for medical decisions, focussing on illness understanding and skilful goals of care discussions.^20^ However, high-quality advance care planning aimed at identifying and achieving preferred place of death cannot be maximised without increasing the number of available services, funding and support for those with terminal illnesses who wish to die at home.^21^ Studies in oncology have shown that palliative care referral more than 3 months prior to death is associated with fewer hospitalisations and ED presentations in the last 30 days of life^22^ and a lower likelihood of dying in hospital.^23^ While this study did not collect data on palliative care referral dates, the findings demonstrate that of those with a ‘palliative’ care type hospital admission recorded, most were within the last 7 days of life and very few patients had a ‘palliative’ care type hospital admission recorded more than 3 months prior to death. This may be explained by specialist palliative care referral being viewed as end-of-life care^24^ and models of specialist palliative care for heart failure focussing on triggers and specific clinical criteria.^25^

Recently published work to determine valid referral criteria to specialist palliative care showed that most of the major criteria were diseased-based, followed by needs-based.^26^ Many of the disease-based criteria occur later in the illness trajectory and underscores current accepted practices.^26^ The reliance on disease-based criteria is understandable given that they are objective and tangible, and that the trajectory of heart failure is unpredictable.^26^ However, the call for embedding palliative care in heart failure care based on unmet needs is gaining momentum in heart failure guidelines.^27^ An integrated and collaborative model is favoured, with palliative care being provided alongside heart failure management by heart failure nurses, clinicians and allied health professionals trained in palliative care and working as a team, with specialist palliative care involved for more complex unmet needs, throughout the illness.^28,29^ Earlier involvement of specialist palliative care in a collaborative manner has the potential to improve patients’ and carers’ quality of life, deliver care to the whole patient and carer, manage symptoms and coordinate services.^27^ An increase in the number of individuals dying in their preferred place of death and subsequent lower hospitalisation costs are also likely positive outcomes of early, collaborative involvement of specialist palliative care on an as-needed basis.^30^

### Strengths and limitations

The strengths of this study include the use of population-level data considered to be high quality (Queensland Hospital Admitted Patient Data Collection), over a ten-year time frame. The use of the health administrative data provides insight into the age and sex of patients with heart failure, unbiased by recruitment methods used in other study designs. However, health administrative data are not designed from the outset for research purposes. Therefore, there was limited control over the quality of the recorded data and typographical errors are possible due to the use of probabilistic linkage, where the concept of probability is used to produce a composite score when matching a pair of records based on odds ratios. We identified two individuals who had hospital admissions occurring after their date of death, as these likely reflect typographical errors or incorrect linkages. If these were considered false positives, we would equate to <0.1% false positive linkages between the death data and Queensland Hospital Admitted Patient Data Collection records. The data for this study did not include care which occurs in the community, including outpatient care and community based palliative care services which are opportunities to deliver timely palliative care.

## Conclusion

This study highlights the repeated hospital use in the year preceding death, with many older patients dying in hospital, without accessing palliative care. Improving and providing timely palliative care, including end-of-life care, is needed to reduce avoidable hospitalisations and address symptom burden. This study provides foundational evidence for future research on the development of services for individuals with heart failure in their last year of life.
